# Effectiveness and Feasibility of a Remote Lifestyle Intervention by Dietitians for Overweight and Obese Adults: Pilot Study

**DOI:** 10.2196/12289

**Published:** 2019-04-11

**Authors:** Karin Haas, Stefanie Hayoz, Susanne Maurer-Wiesner

**Affiliations:** 1 Applied Research & Development Nutrition and Dietetics Department of Health Professions Bern University of Applied Sciences Bern Switzerland; 2 Center of Obesity and Metabolism Winterthur GmbH Winterthur Switzerland

**Keywords:** remote consultation, obesity, weight loss, mobile app, behavior therapy, healthy lifestyle, healthy diet

## Abstract

**Background:**

To tackle the problem of obesity and related diseases in Switzerland, cost-efficient, effective, and innovative primary health care interventions for weight management are required. In this context, Oviva has developed a scalable technology for registered dietitians to counsel overweight and obese patients via a mobile phone app.

**Objective:**

The aim of this study was to evaluate the effectiveness and feasibility of weight loss counseling by dietitians using a mobile phone app for patients with overweight and obesity.

**Methods:**

In this pre- and posttest pilot study, overweight and obese adults participated in a 1-year behavioral intervention to lose weight through remote counseling by dietitians in the German-speaking part of Switzerland. The study started in April 2016 and finished in May 2018. Participants received individual counseling through the app and the exchange with the dietitian focused on regular feedback on photo-based food log, motivation, and education. The contents were tailored to the individual lifestyle goal set. The predefined intensity of remote counseling decreased during the year. Group chat could be used. The outcomes examined were changes in weight (primary outcome), hemoglobin A_1c_, fasting glucose, fasting insulin, triglyceride, high-density lipoprotein cholesterol, blood pressure (BP), body mass index (BMI), waist circumference, body fat, and responses to a self-administered questionnaire with questions regarding participants’ physical activity, dietary assessment, and health-related quality of life. Changes were tested at baseline, after 3 months, and after 12 months, as well as between the third and the 12th month.

**Results:**

In total, 36 women and 7 men, with a mean age of 40.6 years, participated and 36 participants completed the study. Median weight change after the first 12 weeks was −3.8 kg (range: −15 to 2.4 and *P*<.001), between week 12 and week 52 it was −1.1 kg (range: −9.7 to 7 and *P*=.08), and the median change during the entire period of intervention was −4.9 kg (range: −21.9 to 7.5 and *P*<.001). Furthermore, changes in BMI, waist circumference, body fat, and BP between baseline and 12 weeks and between baseline and 52 weeks were also significant. Significant changes in certain eating habits were also demonstrated (higher frequency of vegetable, fruit, and breakfast consumption and lower frequency of alcohol, sweet, and fat consumption).

**Conclusions:**

In addition to the professional skills of a dietitian, a profession-specific app such as Oviva can provide effective support that meets the needs of dietitians and clients on the long path of behavioral change and sustainable weight reduction.

**Trial Registration:**

ClinicalTrials.gov NCT02694614; https://clinicaltrials.gov/ct2/show/NCT02694614 (Archived by WebCite at http://www.webcitation.org/76gYkGOIc)

## Introduction

### Background

Obesity has become one of the biggest public health challenges in recent decades [1]. In Europe, more than half of the population is classified as overweight or obese [2]. The prevalence is also high in Switzerland. Currently, 41.6% and 13.9% of men and 19.7% and 11.3% of women are overweight or obese, respectively [3]. Both overweight and obesity are associated with the incidence of noncommunicable diseases including type 2 diabetes, cardiovascular diseases, certain types of cancers, as well as premature death [4,5]. Overweight and obesity are also associated with psychosocial problems such as disordered eating, depression, anxiety, body image dissatisfaction, and low self-esteem in relation to weight stigma [6]. In addition to the negative effects on individual health, obesity is responsible for a large proportion of cost for the health care system and society [7,8]. In 2012, the direct costs of overweight and obesity associated diseases represented 7.2% of the total Swiss health care expenses [9].

International guidelines recommend a weight loss of at least 5% for the treatment of individuals with overweight or obesity to achieve positive clinical effects. The magnitude of weight loss is associated with dose-effect improvements in cardiovascular risk factors such as high blood pressure (BP), hyperlipidemia, and hyperglycemia [10]. In addition, waist circumference and body composition (maintenance or increase in muscle mass and decrease in fat mass) must be considered as important outcomes [11]. To achieve sustainable positive changes in these parameters, the first step must be a lifestyle modification concerning eating behavior and physical activities [10,11]. Behavior-based interventions have been shown to be effective for weight loss outcomes including change in weight and waist circumference [12,13]. In addition, the effectiveness of a weight loss program may be influenced by the intensity of an intervention and the continuous provision of a varied program [10,14]. However, behavior change requires time and must be maintained. At this maintenance period, numerous barriers, including the absence of social support, a lack of time management, health status changes, life transitions, and lack or decline of motivation can impact on the original success of weight loss [15,16].

Therefore long-term, cost-efficient, effective, and innovative primary health care interventions for weight management are needed.

### Web-Based Nutrition Counseling

New digital technologies could be a possible solution as they provide innovative counseling possibilities with greater flexibility to coach overweight and obese clients. Such technologies can help reach clients who are unable and/or unwilling to engage in face-to-face counseling and make it possible to assist clients more conveniently and as required (eg, in the weight maintenance phase). Studies show that both face-to-face and remote counseling are effective methods for losing weight, as well as for maintaining weight loss [17-20]. Mobile phone app and short message service (SMS) text messaging could offer effective intervention strategies in this context. For example, SMS text messaging is a cheap, portable, convenient, and innovative medium facilitating goal setting, self-monitoring, and information exchange [21] and allows individualization, which are important components of behavior change. For obese or overweight adults, personalization through counseling, individualized feedback, as well as social support and self-monitoring system seem to be important when using mobile phones to assist behavior change and therefore promote weight loss [22,23]. Small successes can be visualized with these tools, which may have an impact on patient self-efficacy. This may in turn support further changes in behavior and lead to long-term successful weight management [24].

Although these innovative tools offer dietitians new potential for counseling and supporting eating and physical behavior change, their use of these tools remains limited. One reason may be that few studies on the long-term impact of Web-based nutrition counseling exist. The number of commercial health and nutrition apps available is huge; however, specific apps for remote counseling, which are aligned to the needs of a dietitian, are limited. In Switzerland, Oviva, a new type of health care provider, has come to the market using new technology to scale the dietitian workforce. Oviva has developed a scalable technology for dietitians to counsel overweight and obese patients via a mobile phone app. Through this app, dietitians can organize their sessions through messaging and video calls, have access to photo food diary, activity, and weight tracking features allowing them to monitor client progress through their photos, activity, and weight logs, as well as set goals and share documents and surveys. Assessing the implementation of this novel kind of intervention is important [25,26] as similar innovative solutions for use by dietitians have not yet been investigated in Switzerland. The aim of this pilot study was to evaluate the effectiveness and feasibility of weight loss counseling using the mobile phone app Oviva for overweight or obese adults.

## Methods

### Study Design

This study was carried out as a 1-year, single arm pre- and postpilot intervention with overweight or obese adults in Switzerland from March 2016 (first participant in) to May 2018 (last participant out). All study procedures were approved by the local institutional review board and the study is registered under ClinicalTrials.gov (NCT02694614). Follow-up will be conducted 1 year after the intervention.

### Study Population and Recruitment

Sample size was calculated with a significance level of 0.05 and power of 80%. A sample size of 36 was needed to detect a weight loss of 0.5 SD, which corresponds to a medium effect size according to Cohen, using a 2-sided Wilcoxon signed rank test. To account for dropouts, 50 participants were planned. After screening, 43 participants were included in the study.

Participants were included in the study if they met the following inclusion criteria: adults (aged 18 years and over), body mass index (BMI) between 26 and 33 kg/m^2^, fluent in German, mobile phone user (iOS or Android), and capable of sending and receiving SMS text messages and pictures. Participants were excluded if they were pregnant or breastfeeding, were diagnosed with conditions other than dyslipidemia, hypertension, and insulin resistance requiring nutrition therapy, had serious disease requiring continuous drug therapy, were on a weight reduction diet during the last 6 months, took medication for weight loss in the past, or enrolled in another weight loss program. Subjects were invited to participate with flyers distributed through the Center for Obesity and Metabolism Medicine Winterthur (in Canton Zurich), via general practitioners, advertisements on the websites of the participating research institutions, local newspapers, and through word of mouth advertising. Interested persons received written and verbal information about the study. Screening took place following informed consent to determine the eligibility of participants on the basis of the inclusion and exclusion criteria at the Center for Obesity and Metabolism Medicine Winterthur.

### Oviva App

**Figure 1:**
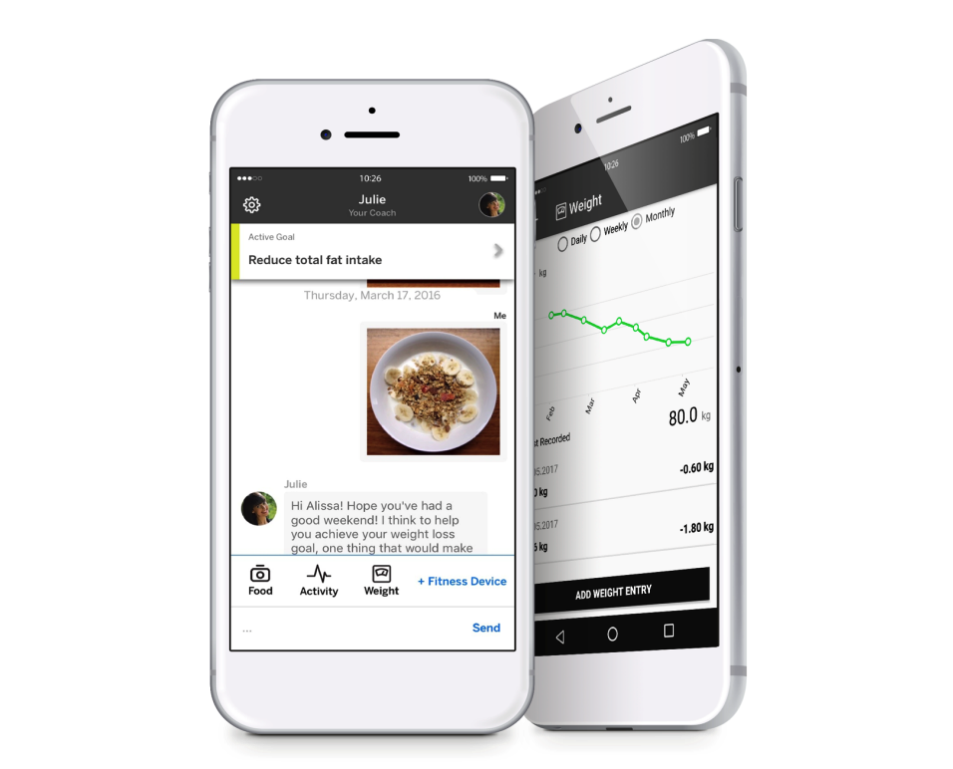
Client view of a chat communication including a photo-based food log and weight log.

The program is designed to facilitate personal coaching by registered dietitians. This digital communication system connects patients remotely with their dietitian who has all the tools required for remote counseling. Via customer-facing apps for iOS and Android, all clients are able to collect relevant information (eg, food intake, physical activity, and weight) and communicate easily with their dietitian. All features of the app are modeled on typical activities in a dietitian’s everyday practice and include chat-like communication with dietitians, group chats for support from peers, dietitian’s profile to create a more personal connection, a photo-based food log, activity and weight logs, a goal scorecard, showing past goals and future options, a content database, feedback to the dietitian, and links to standard learning materials. Client exchanges with the dietitian focused on regular feedback regarding the photo-based food log, motivation, and education (eg, recipes, nutritional facts, and challenges). Clients were also able to join group sessions within the same app. All data were transferred and saved via secure channels. For the dietitians, all information was displayed in a secure cloud-based platform, similar to Webmail. They could review all relevant information for each client individually, take notes, create customized surveys, and use a content database, including relevant links. Figure 1 shows the client view of a chat communication including a photo-based food log and weight log.

### Intervention

This behavior-based intervention was carried out entirely remotely by 3 registered dietitians using Oviva technology. The intervention comprised 3 different coaching phases as described in Textbox 1.

The content of the remote counseling was tailored to the specific needs of each participant, but it was tailored with the overall aim to lose weight. The behavioral strategies used were self-monitoring, goal setting, stimulus control, elaboration of alternative strategies, social support, positive reinforcement, and relapse prevention (particularly toward the end of the intervention). Motivational interview techniques were used throughout the entire intervention process.

Key counseling topics were based on the needs of the participants and included negative energy balance, plate model (Swiss tool for the correct proportions of each food group at every meal), portion size, meal rhythm, energy density of food, quality of carbohydrate, fat, carbohydrate-modified diet, adequate protein sources, dealing with special situations (such as emotional eating, invitations to dinner and parties, holidays, eating triggers, lapses), grocery shopping, and physical activity.

### Outcome Measures: Anthropometric and Metabolic Risk Factors

The primary outcome was defined as weight change between baseline (M0) and 1 year (M12). All measurements were examined at baseline (M0), at 3 months (M3), and 1 year (M12), and it included anthropometric, clinical, and questionnaire assessments. Anthropometric and clinical assessments included body height, body weight, BMI, waist circumference, body fat, hemoglobin A_1c_ (HbA_1c_), fasting glucose, fasting insulin, triglyceride, high-density lipoprotein (HDL) cholesterol, and BP. Measurements were carried out by a clinical nurse in the morning. Participants were instructed to fast overnight (8 hours), abstain from smoking, consuming alcohol, and drinking caffeine-containing beverages, as well as from vigorous exercise 48 hours before blood samples were taken and body composition was measured. Weight was measured using a clinically validated and calibrated scale (Seca mBCA 515, medical Body Composition Analyzer). Participants were lightly dressed, without shoes, and weight was recorded to within 0.01 kg. Height was measured using a calibrated stadiometer (Forma Seca). The measurement was carried out in an upright position with feet positioned on the floor board of the stadiometer. Heels, buttocks, and the back of the head touched the back board of the stadiometer with arms on the sides. BMI was calculated by dividing body weight in kilograms by height in meters squared. For the measurement of the waist circumference, an inelastic tape was placed directly on the skin between the lower rib and iliac crest. Each measurement was performed twice after exhalation and recorded within 0.5 cm. If the difference between the 2 measurements was greater than 1 cm, a third measurement was performed and the mean of the 2 closest measurements was calculated. Body composition was measured by bioelectrical impedance analysis (Seca mBCA 515, medical Body Composition Analyzer) with participants in upright position, lightly dressed, and barefoot. BP measurements were performed using the Riva-Rocci method with a special instrument (Firma boso-medicus SN 768 00 440 729 Bosch+Sohn GmbH und Co) and a stethoscope. The measurement was performed 3 times using the right arm after participants had been sitting for at least 10 min. The average of these measurements was used for analysis. Blood samples were obtained after overnight fasting from the antecubital vein to determine HbA_1c_, fasting glucose, fasting insulin, triglyceride, and HDL cholesterol. All laboratory assays were performed by Labor Risch Schaffhausen using standardized methods as described in the study protocol.

**Phases of intervention.**

Prephase or initiation (week −2 to 0)

- Complete initial assessment; implementation: photo-assisted dietary and activity recording; setting overall aim.

Phase 1: Transition (month 1 to 3)

- Mobile phone assisted patient coaching 5 times per week; feedback on nutrition and activity; setting 1 or 2 specific goals for 2 weeks at the time; assessing goals and adapting them if necessary; providing information and education materials that are appropriate to the goals; 1 Skype call at the end; clinical assessment at the end.

Phase 2: Stabilization (month 4 to 6)

- Mobile phone assisted patient coaching 3 times per week; feedback on nutrition and activity; strengthening new behavior; setting new goals if necessary; assessing goals and adapting them if necessary; providing information and education materials that are appropriate to the goals; exchange with peer groups (optional and anonymous, coach guided).

Phase 3: Maintenance (month 7 to 12)

- Mobile phone assisted patient coaching once every 2 weeks; feedback on nutrition and activity if required; exchange with peer groups (optional and anonymous, noncoach guided or coach guided); access to Web-based education materials; final chat; clinical assessment at the end.

### Additional Outcome Measures

A questionnaire was used to assess socioeconomic data, dietary assessment, physical activity, and health related quality of life. Physical activity was assessed using the self-administered Global Physical Activity Questionnaire (GPAQ), developed and validated by the World Health Organization. Dietary intake was investigated using a brief 11-item simplified food frequency questionnaire. The English version of this questionnaire was developed and tested in a diverse population as a relatively simple, valid, and efficient tool for dietary assessment [27]. The English version was translated into German, tested for comprehensibility in a pretest and adapted. The questionnaire includes questions on the following categories: fast food and convenience foods, fruits, vegetables, sweetened beverages, unsweetened beverages, alcoholic beverages, salty snacks, sweets and desserts, fat for food preparation, fatty spreads, and breakfast consumption. Response options for the items are organized into 3 response categories: first category represents the most healthful dietary consumption (0 points), second category represents less healthful consumption (1 point), and the last category represents the least healthful consumption (2 points). The total score (out of 22 points) provides an indication of how healthy the diet is, with a low score indicating a healthy diet. Quality of life was assessed using the 12-item Short-Form Health Survey (SF-12; short version of SF-36, instrument for assessing health-related quality of life in obesity research), which includes 12 items covering physical functioning, role limitations because of physical health problems, bodily pain, general health, energy or fatigue, social functioning, and role limitations because of emotional problems and mental health [28]. The SF-12 generates a physical and a mental health component summary score (PCS and MCS) with a total maximum of each score of 100 (higher scores indicate higher quality of life). The SF-12 scores compare favorably with those obtained using the SF-36 [29].

### Statistical Analysis

All outcomes were analyzed on the basis of the intention-to-treat (ITT) population, defined as all participants who started the intervention. Supportive analyses based on the per-protocol (PP) population were performed for selected outcomes. For all outcomes, the changes were calculated from baseline to the specified time points. The changes over the whole observation period were investigated using nonparametric analysis of variance methods for longitudinal data [30]. Posthoc tests for changes at all measured time points were performed using Wilcoxon signed rank tests. Two-tailed tests with significance level .05 were performed for all analyses. No adjustment for multiple testing was performed. All outcome variables were summarized using descriptive statistics. All analyses were performed using R version 3.4.3 (R Foundation for Statistical Computing).

## Results

### Study Population

Following screening, a total of 43 people aged between 20 and 67 years were included in the study. 36 participants completed the 1-year intervention. Overall, the proportion of women was higher (84%, 36/43) than men, with 6 women and 1 man not completing the intervention. Socioeconomic data of the study population are summarized in Table 1.

In total, 33 people were initially diagnosed with dyslipidemia, 17 with insulin resistance, and 4 with hypertension. No participant was diagnosed with diabetes and 1 participant was diagnosed with prediabetes because of a slightly elevated HbA_1c_ level. At the beginning of the study, 3 people took medication to treat hypertension and 2 people took lipid-lowering medication. The medication did not change during intervention.

### Outcome Measures: Anthropometric

**Figure 2:**
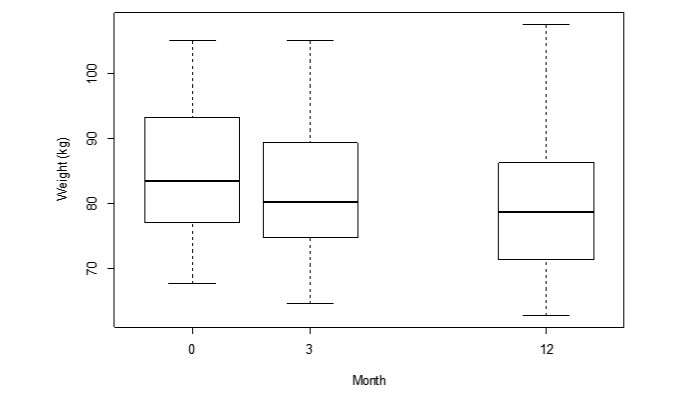
Median weight (in kg) at different time points: month 0 (N=43), month 3 (N=40), and month 12 (N=36).

The results are reported as median values unless otherwise indicated. The median weight at different timepoints (M0: 83.5 kg, M3: 80.3 kg, and M12: 78.7 kg) are shown in Figure 2. The weight loss between baseline (M0) and month 12 (M12) was −4.9 kg or 6% (*P*<.001). Weight loss was also significant between M0 and M3 (−3.8 kg, *P*<.001) but not between M3 and M12 (−1.1kg, *P*=.08; Table 2). Overall, 58% (21/36) of participants achieved a weight loss of 5% or more from their initial weight, and 5 participants gained weight (between 2.1 kg and 7.5 kg). Of the 5 participants who gained weight during the total intervention, 4 also lost no weight in the first 3 months.

The BMI decreased significantly between M0 and M12 (by 1.8 kg/m^2^ [*P*<.001]) and between M0 and M3 (by 1.4 kg/m^2^ [*P*<.001]). Between M3 and M12, the decrease was 0.4 kg/m^2^ (*P*=.06). Waist circumference, body fat (kg), or body fat (%) showed similar trends; a significant reduction between M0 and M12 (*P*<.001), as well as between M0 and M3 (*P*<.001), but no significant effects between M3 and M12 (waist circumference *P*=.72, body fat [kg] *P*=.14, body fat [%] *P*=.20; Table 2). Percentage changes in weight, BMI, and fat (%) are shown in Table 3.

**Table 1:** Socioeconomic data (N=43).

Characteristics		Statistics
Women, n (%)		36 (84)
Age (years), mean (SD)		40.6 (12.4)
**Marital status,** **n (%)**		
	Married	18 (42)
	Single	15 (35)
	Divorced	6 (14)
	Separated	3 (7)
	Widowed	1 (2)
**Children living in the household, n (%)**		
	0	19 (44)
	1	9 (21)
	2	11 (26)
	3	4 (9)
**Net household income per month, n (%)**		
	Less than 3000 CHF^a^	1 (2)
	3000 to 4500 CHF	6 (14)
	4500 to 6000 CHF	6 (14)
	6000 to 9000 CHF	13 (30)
	More than 9000 CHF	17 (40)
**Highest level education,** **n (%)**		
	Compulsory education	0 (0)
	Apprenticeship or Matura	19 (44)
	Universities or Universities of Applied Sciences or Higher Vocational Education	24 (56)
**Nationality, n (%)**					
	Swiss	36 (84)
	Other (German, Great Britain, United States, Austria, and Italy)	7 (16)

^a^CHF: Swiss Francs.

### Outcome Measures: Other Metabolic Risk Factors

Weight loss observed among different timepoints appeared to have no effect on glucose metabolism (HbA_1c_, blood glucose, and insulin) and blood lipids (triglyceride and HDL cholesterol). In contrast, a significant reduction in systolic BP was observed among all timepoints (−11.2 mm Hg between M0 and M12, *P*<.001), as well as in diastolic BP between M0 and M12 (−5.5 mm Hg, *P*<.001; see Table 2). The supportive analyses based on the PP population showed similar results to the ITT analysis. The results of anthropometric measurements and other metabolic risk factors at baseline (M0), 3 months (M3), and 12 months (M12) are presented in Multimedia Appendix 1.

### Additional Outcome Measures

The SF-12 score (MCS and PCS) decreased during intervention, especially during the first 3 months (PCS: 53 to 55.2 and MCS: 53.2 to 54.9), but the changes were not significant (PCS: *P*=.08 and MCS: *P*=.09). The values for health-related quality of life at month 12 (PCS: 55.4 and MCS: 55.1) showed little change in comparison with the values at month 0 and the changes were also nonsignificant (PCS: *P*=.58 and MCS: *P*=.58). The overall result of GPAQ showed a small but nonsignificant increase in total moderate-vigorous physical activity metabolic equivalent minutes per week (MET-min/week) among different assessment times (median M0: 1920, M3: 2360, M12: 2740; M0-M3: *P*=.06, M3-M12: *P*=.78, M0-M12: *P*=.15). An analysis according to the 3 subcategories showed the following results. In subcategories *activity at work / travel to and from places,* no changes were apparent over the entire course of the year, whereas there were significant changes in the category *recreational activities* during this period (M0: 960 MET-min/week, M12: 1700 MET-min/week; M0-M12: *P*=.007). The results for certain eating habits (higher frequency of fruit, vegetable, and breakfast consumption and lower frequency of alcohol, sweet, and fat consumption) were significant between M0 and M3 and M0 and M12 and for salty snacks between M0 and M3. The total score for dietary consumption (median M0: 6 points, M3: 4 points, M12: 4 points) showed a significant reduction respective improvement toward a healthier diet between M0 and M3 (*P*<.001) and M0 and M12 (*P*<.001).

**Table 2:** Changes in anthropometric measurements and other metabolic risk factors between baseline (M0) 3 months (M3), and 12 months (M12).

Outcomes		Median	Range (min-max^a^)	*P* value
**Weight (kg)**				
	Difference between M0 and M3 (N=40)	−3.8	−15 to 2.4	<.001
	Difference between M3 and M12 (N=36)	−1.1	−9.7 to 7	.08
	Difference between M0 and M12 (N=36)	−4.9	−21.9 to 7.5	<.001
**Body mass index (kg/m^2^)**				
	Difference between M0 and M3 (N=40)	−1.4	−4.5 to 1.1	<.001
	Difference between M3 and M12 (N=36)	−0.4	−3.1 to 2.3	.06
	Difference between M0 and M12 (N=36)	−1.8	−6.9 to 2.5	<.001
**Waist circumference (cm)**				
	Difference between M0 and M3 (N=40)	−3.5	−23 to 5	<.001
	Difference between M3 and M12 (N=36)	0	−6.6 to 13.7	.72
	Difference between M0 and M12 (N=36)	−3.8	−17.8 to 9	<.001
**Body fat (kg)**				
	Difference between M0 and M3 (N=40)	−3.3	−10.6 to 2.5	<.001
	Difference between M3 and M12 (N=36)	−0.6	−7.4 to 7.4	.14
	Difference between M0 and M12 (N=36)	−4.0	−16.9 to 6.4	<.001
**Body fat (%)**				
	Difference between M0 and M3 (N=40)	−2.3	−7.6 to 2.5	<.001
	Difference between M3 and M12 (N=36)	−0.3	−9.9 to 5	.20
	Difference between M0 and M12 (N=36)	−2.5	−11.9 to 3.7	<.001
**Hemoglobin A_1c_ (%)**				
	Difference between M0 and M3 (N=40)	0	−0.5 to 0.3	.36
	Difference between M3 and M12 (N=36)	0	−0.6 to 0.4	.84
	Difference between M0 and M12 (N=36)	0	−0.5 to 0.5	.08
**Blood glucose (mmol/L)**				
	Difference between M0 and M3 (N=40)	0	−1 to 0.6	.44
	Difference between M3 and M12 (N=36)	0	−1.2 to 0.8	.66
	Difference between M0 and M12 (N=36)	0	−1 to 1	.05
**Insulin (mlU/L)**				
	Difference between M0 and M3 (N=40)	−1.35	−12 to 10.5	.39
	Difference between M3 and M12 (N=36)	−0.15	−12.7 to 33.2	.69
	Difference between M0 and M12 (N=36)	−1.35	−9.1 to 37.7	.33
**Triglyceride (mmol/L)**				
	Difference between M0 and M3 (N=40)	−0.14	−2.09 to 1.81	.13
	Difference between M3 and M12 (N=36)	0.01	−2.38 to 0.99	.61
	Difference between M0 and M12 (N=36)	−0.14	−2.46 to 1.33	.07
**High-density lipoprotein cholesterol (mmol/L)**				
	Difference between M0 and M3 (N=40)	−0.06	−0.64 to 0.3	.09
	Difference between M3 and M12 (N=36)	0.11	−0.31 to 0.55	.01
	Difference between M0 and M12 (N=36)	0.02	−0.32 to 0.78	.63


**Blood pressure systolic (mm Hg)**				
	Difference between M0 and M3 (N=40)	−6.2	−28.4 to 20.7	.003
	Difference between M3 and M12 (N=36)	−6.4	−31.3 to 22.3	.05
	Difference between M0 and M12 (N=36)	−11.2	−29 to 10.3	<.001
**Blood pressure diastolic (mm Hg)**				
	Difference between M0 and M3 (N=40)	−1.8	−22 to 13.7	.16
	Difference between M3 and M12 (N=36)	−1.4	−26.3 to 16	.16
	Difference between M0 and M12 (N=36)	−5.5	−20.3 to 12	<.001

^a^min-max: minimum-maximum.

**Table 3:** Percentage changes between baseline (M0), 3 months (M3), and 12 months (M12) in weight, body mass index, and body fat percentage.

Measurements		Median	Range (min-max^a^)
**Weight**			
	Percentage difference between M0 and M3 (N=40)	−4.6	−15.6 to 3.3
	Percentage difference between M3 and M12 (N=36)	−1.5	−10.7 to 7.9
	Percentage difference between M0 and M12 (N=36)	−6.0	−21.3 to 8.6
**Body mass index**			
	Percentage difference between M0 and M3 (N=40)	−4.8	−15.6 to 3.9
	Percentage difference between M3 and M12 (N=36)	−1.4	−10.7 to 7.9
	Percentage difference between M0 and M12 (N=36)	−6.2	21.3 to 8.6
**Body fat percentage**			
	Percentage difference between M0 and M3 (N=40)	−5.4	−25.5 to 6.8
	Percentage difference between M3 and M12 (N=36)	−0.9	−23.9 to 13.7
	Percentage difference between M0 and M12 (N=36)	−6.5	−35.8 to 9.8

^a^min-max: minimum-maximum.

## Discussion

### Principal Findings

This study investigated whether a 1-year remote counseling intervention by dietitian targeting weight loss was effective and feasible for people with overweight and obesity. The results show that this form of behavior counseling leads to significant weight loss, both in the first 3 months during an intensive remote counseling period, as well as over the entire course of 1 year. Weight loss also occurred between the third and 12th month, although this was not significant. The positive results in this study are in line with other studies investigating Web-based counseling and weight loss [17,31-33]. In 1 study, participants receiving remote support by weight loss coaches attained a mean (SD) change in weight from baseline of −4.6 (0.7) kg after 24 months, with weekly calls during the first 3 months and after that, with 1 call each month [17]. In a pilot randomized controlled trial with the aim to evaluate the feasibility and acceptability of a Web-based weight loss intervention in men with type 2 diabetes with feedback from a dietitian and exercise expert, the median weight loss was −4.3 kg (−7.8 to −1.0) from baseline to 12 months [31]. Other Web-based interventions that have focused on weight loss with shorter duration have demonstrated positive results [32-35]. For example, 1 study tested the effectiveness of a behaviorally-based mobile phone app combined with SMS text messaging from a health coach (participants could choose the frequency) and demonstrated significant weight loss after 3 months in the group with both mobile phone and health coach (−1.8 kg, *P*=.01) [33]. However, these interventions were only performed for 3 months and lack a long-term follow-up measurement. Changes in other anthropometric parameters associated with cardiovascular risk, including BMI, body fat percentage, and waist circumference were also significant in this study, similar to other remote interventions [32,33]. However, a direct comparison is difficult because of methodological differences such as duration, intensity, and frequency of counseling sessions among interventions. In addition, communication took place via different channels, such as SMS text messages, emails, phoning, and on the Web, and with counsellors who had different qualifications.

Over half of the participants lost at least 5% of their initial weight. This amount of weight loss is typically associated with positive clinical effects, namely an improvement in cardiovascular risk factors. A systematic review demonstrated the following effects of dietary advice: reduction in systolic BP by 2.61 mm Hg (95% CI 1.31-3.91), diastolic BP by 1.45 mm Hg (95% CI 0.68-2.22), and 24‐hour urinary sodium excretion by 40.9 mmol (95% CI 25.3-56.5) after 3 to 36 months, between intervention and control group. However, no changes in HDL cholesterol, triglyceride levels, and fasting glucose were seen [36]. Although group comparisons were made in contrast to this pre- and postsingle arm study, the results indicate a similar trend. This study’s results showed significant reduction in systolic BP and diastolic BP, but no significant reduction was seen in triglycerides, HDL cholesterol, HbA_1c_, blood glucose, and insulin despite weight loss. One reason for this could be the small sample. The sample size calculation was based on the primary outcome. In addition, for some participants, the initial values of these parameters, particularly HbA_1c_, were already within a normal range; therefore, a change in these values was not expected. Furthermore, the initially diagnosed cardiovascular risk factors (dyslipidemia or/and insulin resistance) in many participants could also have an impact on these results, although participants were free of serious diseases and only 5 participants took medication to treat hypertension and/or lipid-lowering medication during intervention.

Weight loss and improvement in other parameters were achieved by lifestyle intervention. The most important components of such weight loss interventions include a moderate calorie-reduced diet, increased physical activity, and the application of behavioral strategies to facilitate compliance with nutrition and activity recommendations [10]. In this study, the diet was analyzed with a very short frequency questionnaire to minimize the burden on participants. A qualitative analysis of individual food groups did not take place and energy intake was not measured. Nevertheless, positive changes were observed for most food groups and included increased intake of fruits and vegetables and lower intake of fat, sweets, and alcohol. These changes in food consumption would have resulted in lower energy intakes and consequent weight loss. An evaluation of the photo diaries and physical activity data available on the mobile phone were not conducted in the context of this study. However, this might lead to additional interesting findings. The measurement on movement behavior also indicated positive effects, particularly moderate and vigorous-intensity sports, fitness or recreational (leisure) activities in the *recreational activities* subcategory, whereas no changes were observed in the subcategories *activity at work / travel to and from places*. Increase in recreational activity is important as this form of activity may contribute to the prevention of cardiovascular diseases and cancer [37]. With regard to quality of life, no significant changes were observed for the PCS and MCS. This could be attributed to the very high quality of life of in Switzerland. In a similar study, the scores for MCS or PCS were lower and/or the initial weight higher and improvement mainly took place in the first half of the year [38]. In this study, the largest improvement in MCS and PCS occurred in the first 3 months.

The authors attribute the reasons for the successful weight reduction in this study to the following aspects. Dietitians used a wide range of behavioral change and counseling techniques (eg, self-monitoring, goal setting, relapse prevention, and motivational interviewing) and applied evidence-based nutritional knowledge according to individual needs (eg, energy density, meal rhythm, quality of carbohydrate, and adequate protein sources). They also provided simple recommendations for physical activity. The results of other studies demonstrate that interventions aimed at changing eating habits or sedentary lifestyle are more effective if they include behavioral change techniques [39,40]. Another review has confirmed the effectiveness of dietetic counseling for adults (not Web-based counseling) aimed at improving diet quality, diabetes, and weight loss outcomes [41].

The intensity of the intervention typically affects study outcomes [14]. In this study, the intensity of the intervention in the first half of the year might also have had an important impact (5 times per week in the first 3 months and 3 times per week during the following 3 months; average duration of 5 min per contact). Staying in contact with participants in the second half of the year to prevent relapses to maintain weight or further reduce it may also explain the successful weight reduction in this study [42]. Furthermore, a good working relationship with health care professionals is described as an important factor in long-term weight management [43]. Therefore, apps such as Oviva have the potential to play an important role in maintaining the relationship between client and dietitian and act as a simple means of supporting weight loss. In a Web-based counseling intervention for overweight or obese people, many effective components of a traditional face-to-face counseling session can be used. In addition, the app Oviva represents a simple and time-saving method of communicating eating behavior to the dietitians. Remote counseling can reduce barriers, such as lack of time, geographical distance, inconvenience, and embarrassment, that prevent possible clients from attending a face-to-face counseling session. This technique should also allow people with lower socioeconomic status to be reached. This is especially important as the prevalence of overweight and obesity is higher in this population group [3]. Although we tried to reach people with lower education levels through the obesity center, most people recruited to this study had undertaken higher education. The needs of people with lower socioeconomic status need to be better understood to facilitate access to such innovative and low-threshold solutions for weight loss.

### Limitations

This study was a 1-arm pilot study; therefore, conclusions concerning effectiveness are limited. Generalization is limited because of the small sample size, individual focus in counseling, and the fact that it was a single-center trial. Randomized control trials are needed to examine efficacy and cost-effectiveness of Oviva and remote counseling in general. Pilot studies are described in the literature as an important first step in assessing the implementation of a novel kind of intervention [25,26]. As similar innovative solutions for use by dietitians have not yet been investigated in Switzerland or are rarely available, this pilot study was an initial milestone. By comparison, in the United States, for example, such tools have increasingly found their way into the everyday working life of dietitians [44].

For this study, it was important to ensure that the counseling process took place in a real-life setting. For example, the study physician held a brief motivating discussion during the clinical assessment at M3 with the participants about the course of the intervention. This might also have had a positive effect on weight progression. To meet the client's needs, small deviations from the contact frequencies were accepted. However, the tolerable limit was specified in the study protocol. Furthermore, 2 clients requested a change of dietitian as they were dissatisfied. This request was granted to prevent these participants from dropping out of the study. This has been considered in the PP analysis. Nevertheless, this indicates that the working relationship with the dietitian has an important influence on weight loss. In addition to the scientific and practical contents, the working relationship with the dietitian must be developed in a different way in comparison with face-to-face counseling. A qualitative study in which health care professionals were interviewed suggested that it is a challenge to establish and maintain an empathic relationship when conducting electronic health (eHealth) lifestyle coaching compared with face-to-face coaching [43]. As eHealth lifestyle coaching does not allow for facial expressions and gestures, specific competencies and experience in remote counseling are required to ensure that what would otherwise be transmitted in conversation is perceived by the client in the same way as it would in face-to-face coaching.

### Conclusions

A mobile phone app for overweight or obese patients represents a new approach to weight loss counseling and has the potential to make a valuable contribution to tackling this important public health nutrition problem. This study has shown that in addition to the professional skills of dietitians, a profession-specific app such as Oviva can provide effective support that meets the needs of both dietitians and clients on the long path of behavioral change and sustainable weight reduction.

## Appendix

Multimedia Appendix 1 Results of anthropometric measurements and other metabolic risk factors at baseline (M0), 3 months (M3) and 12 months (M12).

## Abbreviations

BMI: body mass index
BP: blood pressure
eHealth: electronic health
GPAQ: Global Physical Activity Questionnaire
HbA_1c_: hemoglobin A_1c_
HDL: high-density lipoprotein
ITT: intention-to-treat
M0: baseline or month 0
M3: 3 months
M12: 12 months or 1 year
MCS: mental health component summary score
MET: metabolic equivalent
PCS: physical health component summary score
PP: per-protocol
SMS: short message service
