# Supplementary material for: Effectiveness and Feasibility of a Remote Lifestyle Intervention by Dietitians for Overweight and Obese Adults: Pilot Study
Source: JMIR Mhealth Uhealth. 2019 Apr 11;7(4):e12289. doi: 10.2196/12289 (PMC6482396; doi:10.2196/12289)
Supplement: Multimedia Appendix 1 [file mhealth_v7i4e12289_app1.pdf]

Multimedia Appendix 1. Results of anthropometric measurements and other metabolic risk factors at baseline (M0), 3 months (M3) and 12 months (M12)

	Baseline (M0)	At 3 months (M3)	At 12 months (M12)
	n=43	n=40	n=36
**Weight (kg)**			
Median	83.5	80.3	78.7
Range (min, max)	67.7, 105	64.5, 105	62.8, 107.5
**BMI (kg/m^2)**			
Median	30.2	28.4	28
Range (min, max)	26.4, 33	24.3, 33.5	24.1, 33.5
**Waist circumference (cm)**			
Median	92	85.9	86.5
Range (min, max)	74, 112	73.3, 108	78.5, 110.5
**Body fat (kg)**			
Median	32.6	30.6	29.2
Range (min, max)	25.9, 45	18.1, 46.1	17.2, 43.3
**Body fat (%)**			
Median	40.5	39.0	37.9
Range (min, max)	27.8, 48.5	22.2, 45	21.3, 46.9
**HbA1c (%)**			
Median	5.2	5.1	5.2
Range (min, max)	4.7, 5.9	4.6, 5.8	4.6, 5.8
**Blood glucose (mmol/L)**			
Median	6	5.8	6
Range (min, max)	5, 7.4	4.8, 7.2	4.8, 7.2
**Insulin (mIU/L)**			
Median	9	7.2	7.55
Range (min, max)	3.7, 27.2	2.5, 34.5	2.6, 45.4
**Triglyceride (mmol/L)**			
Median	1.12	1.07	0.95
Range (min, max)	0.58, 4.38	0.5, 4.66	0.57, 3.96
**HDL cholesterol (mmol/L)**			
Median	1.56	1.36	1.54
Range (min, max)	0.88, 2.35	0.9, 2.23	0.87, 2.43
**Blood pressure systolic (mm Hg)**			
Median	135.3	130.2	125.2
Range (min, max)	109.3, 159	103.7, 161.3	104.7, 145.6
**Blood pressure diastolic (mm Hg)**			
Median	83.3	83	80.8
Range (min, max)	71, 102	59.3, 106.3	60.3, 99
